# The Prognostic Effects of Ventricular Heart Rate Among Patients With Permanent Atrial Fibrillation With and Without Coronary Artery Disease: A Multicenter Prospective Observational Study

**DOI:** 10.1097/MD.0000000000000920

**Published:** 2015-06-05

**Authors:** Huaibin Wan, Yanmin Yang, Jun Zhu, Xinghui Shao, Juan Wang, Bi Huang, Han Zhang

**Affiliations:** From the Emergency and Intensive Care Center, Fuwai Hospital, National Center for Cardiovascular Diseases, Chinese Academy of Medical Sciences & Peking Union Medical College, Beijing, China .

## Abstract

Heart rate control is important among patients with either atrial fibrillation (AF) or coronary artery disease (CAD). However, the relationship between the ventricular heart rate and adverse outcomes among patients with AF and CAD remains unclear. This study aimed to assess the prognostic effects of ventricular heart rate in patients with permanent AF (permAF) and CAD.

We performed a multicenter, prospective, observational study of patients with AF in China. Patients≥18 years old with permAF were included and divided into a CAD group and a non-CAD group. All patients underwent 1 year of follow-up. The primary outcome was total mortality. Cox proportional hazard models were used to evaluate the relationship between risk factors and the survival rate in the study population.

A total of 852 patients (69.1±12.7 years old, 43.3% male, 44.7% with CAD) were included in the analysis. Patients with CAD were older, were more likely to be male and exhibited higher prevalences of hypertension, diabetes mellitus, LV dysfunction, chronic obstructive pulmonary disease (COPD) and stroke compared with patients without CAD. During the follow-up period, a higher total mortality rate was noted in the CAD group than in the non-CAD group (21.5% vs 15.5%, *P* = 0.023). In the patients without CAD, the lowest quartile (≤76 beats/min) exhibited the best 1-year survival rate; however, in the patients with CAD, the highest quartile (>110 beats/min) exhibited the worst survival rate. Multivariate adjusted Cox analysis indicated that age (HR 1.039, 95% CI 1.025–1.055, *P* < 0.001) and heart rate (*P* = 0.004) were each independently associated with total mortality.

Patients with CAD have more risk factors, and comorbidities and higher mortality rates than patients without CAD. In the patients with permAF without CAD, a ventricular rate of ≤76 beats/minute was associated with the best survival rate; however, among the patients with CAD, no increased mortality was observed unless the heart rate was >110 beats/min.

## INTRODUCTION

Coronary artery disease (CAD) and atrial fibrillation (AF) are common cardiovascular diseases that share many of the same cardiovascular risk factors, including hypertension, diabetes, and obesity.^1,2^ The overall incidence of CAD among patients with AF is relatively high, reportedly ranging from 18.1% to 41.8%,^3–6^ and in patients with CAD, AF is an independent predictor of poor long-term outcomes.^7,8^

Resting heart rate in sinus rhythm is a simple measurement with prognostic implications. A high resting heart rate is a predictor of both total and cardiovascular mortality that is independent of other risk factors among patients with CAD.^9,10^ In patients with AF, a high ventricular heart rate also increases the potential of developing tachycardia-induced cardiomyopathy. Therefore, heart rate control has been recommended as a front-line therapy in the management of most cardiovascular diseases, including CAD and AF.^11,12^ However, previous studies failed to observe improved survival as a result of heart rate control among patients with permanent AF (permAF).^3,13–15^ Moreover, in patients with CAD and AF, whether a lower heart rate correlates with improved survival rate remains uncertain. Therefore, we performed this study to assess the prognostic effects of the ventricular heart rate among patients with permAF and CAD.

## METHODS

### Study Design and Participants

This was a multicenter, prospective, observational study designed to evaluate the clinical characteristics, treatments, and long-term outcomes of patients with AF. Twenty representative centers throughout China (including academic and nonacademic, general and specialized, and urban and rural centers) participated in the study. Patients were recruited from the outpatient clinic of each center between November 2008 and October 2011. All centers were encouraged to enroll consecutive patients. Patients of interest were identified by reviewing clinical records, electrocardiograms, Holter monitor results, and electronic hospital databases and were screened by research staff. Treatment decisions were made at the discretion of the treating physician. All participants provided their written informed consent using materials approved by the ethics committees of each institution and the China National Center for Cardiovascular Disease. The inclusion criteria included an age of 18 years or older and a diagnosis of permAF. The exclusion criteria were acute infectious diseases, a fever of unknown cause, an implanted pacemaker, a diagnosis of severe atrioventricular block, a diagnosis of mental illness, and prior enrollment in another study. Eligible participants were divided into 2 groups according to their CAD history.

### Data Collection

Baseline clinical data were collected, including patients’ demographic information, medical histories, medications, and the primary reasons for and the outcomes of their visits by interviewing the patients, reviewing their medical records, and contacting their treating physicians. Each patient's resting heart rate was measured via 12-lead electrocardiography following at least 3 minutes of rest in the supine position. The left ventricular ejection fraction (LVEF) was measured using Simpson biplane method.^16^ Diagnoses of medical conditions were based on the patients’ clinical records. PermAF was defined as an episode of AF that did not terminate either spontaneously or with electrical or chemical cardioversion, or for which cardioversion was not attempted. Diagnoses of CAD were made in patients with a history of prior myocardial infarction,^17^ prior revascularization (ie percutaneous coronary intervention or prior coronary artery bypass grafting), coronary stenosis ≥50% of the luminal diameter determined by angiography,^18^ or reliable noninvasive imaging evidence of myocardial ischemia (such as coronary computed tomographic angiography, stress myocardial perfusion imaging via either single photon emission computed tomography or positron emission tomography, and ventricular wall motion imaging by stress echocardiography or cardiac magnetic resonance imaging). Heart failure (HF) diagnosis and New York Heart Association (NYHA) classifications were made based on clinical guidelines.^19^

### Follow-Up and Outcomes

Follow-up interviews were conducted via telephone or clinic visits every 3 months until either death or October 2012. The primary outcome was total mortality, including death from vascular and nonvascular causes. The secondary outcomes were systemic embolism and major bleeding. All endpoints were adjudicated via an independent committee that was blinded to the patients, using standardized definitions.

Death from vascular causes was defined as death from either cardiovascular or cerebrovascular insults; the other causes of death were classified as death from nonvascular causes. Systemic embolism included stroke and noncentral nervous system (CNS) embolism. Stroke was defined as the sudden onset of a focal neurological deficit in a location consistent with the territory of a major cerebral artery. Non-CNS embolism was defined as the acute vascular occlusion of an extremity or organ, documented by imaging, surgery, or autopsy. Major bleeding was defined as either fatal bleeding or symptomatic bleeding in a critical area or organ, as well as bleeding causing a fall in hemoglobin ≥20 g/L (1.24 mmol/L) or requiring the transfusion of ≥2 units of either whole blood or red blood cells.

### Statistical Analysis

The categorical variables were expressed as frequencies and percentages, and the normally distributed continuous variables were presented as the means with standard deviations (SDs). Ventricular heart rates were evaluated as continuous variables and as 4 categories divided by quartiles. Comparisons of the categorical data were performed using either the Pearson χ^2^ test or Fisher exact test, and comparisons of the continuous variables were performed using Student *t* test. Both the univariate and multivariate Cox regression models were used to analyze the correlations of risk factors and clinical outcomes, and were restricted to the time of the first event. Hazard ratios (HRs) and 95% confidence intervals (95% CIs) were calculated. To avoid overfitting, variables were only included in the forward conditional stepwise Cox regression analysis if they were significantly associated with outcomes in the univariate analysis (*P* ≤ 0.10). SPSS 16.0 was used to perform the statistical analysis; a *P* value <0.05 was considered statistically significant (2-tailed test).

## RESULTS

We recruited a total of 976 patients with permAF. Of these participants, 64 patients with cardiac implantable pacemakers, 18 patients with severe atrioventricular block, and 42 patients diagnosed with an acute infectious disease at the time of their first clinic visit were excluded. Thus, 852 patients (mean age 69.1 ± 12.7 years old, 43.3% male, 381 [44.7%] patients with CAD) were included in the analysis.

The patients’ basic clinical characteristics are included in Table 1. In contrast to the patients without CAD, the patients with CAD were approximately 10 years older, were more likely to be male, and exhibited higher systolic blood pressures; higher prevalences of hypertension, diabetes mellitus, LV dysfunction (LVEF<45%), chronic obstructive pulmonary disease (COPD), and stroke; and a lower prevalence of valvular heart disease (VHD). The patients with CAD were less likely to have been prescribed digoxin and warfarin but were more likely to have taken aspirin, β-blockers (BBs), angiotensin-converting enzyme inhibitors/angiotensin receptor blockers (ACEIs/ARBs), calcium channel blockers (CCBs) and statins compared with the patients without CAD. The groups were well matched in terms of heart rate, history of HF, and the use of diuretics. As presented in Table 2, the distributions of the different types of CAD were balanced in our study population.

**TABLE 1:**
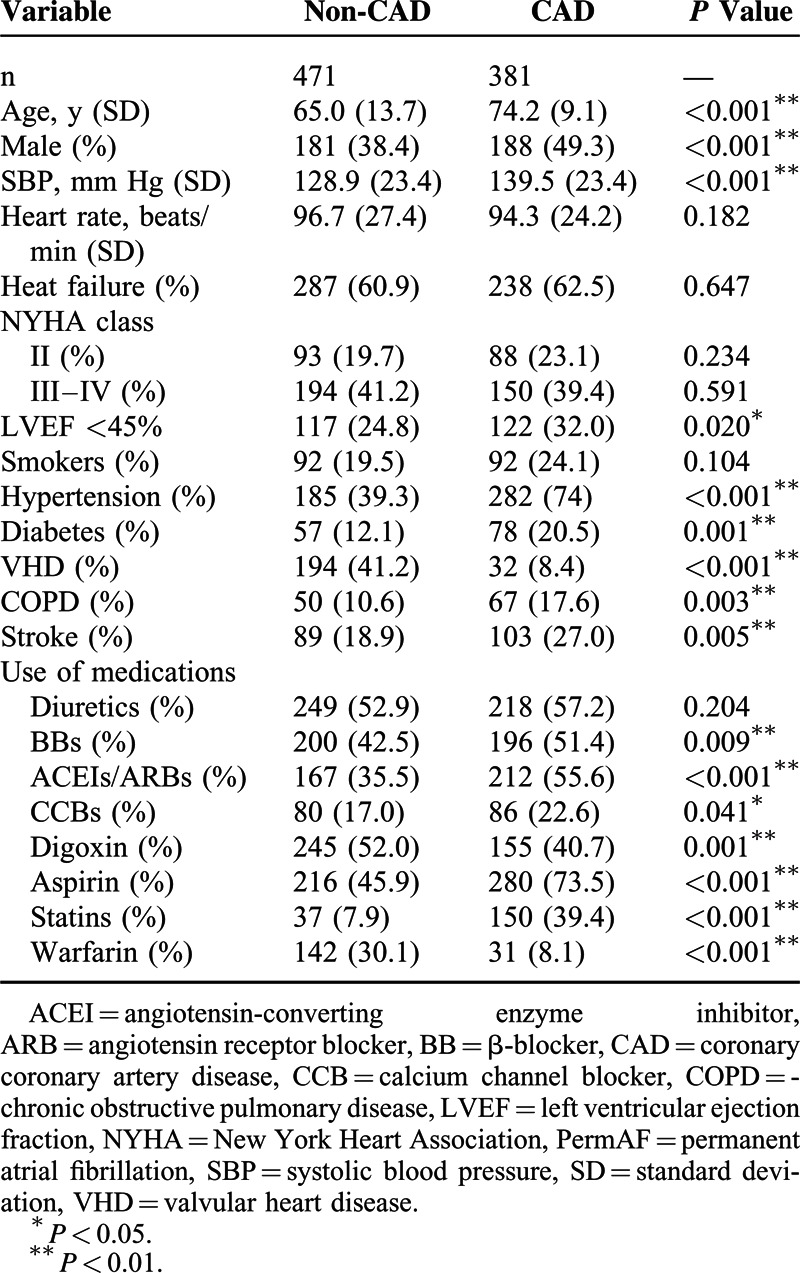
Characteristics of Patients With PermAF at Baseline

**TABLE 2:**
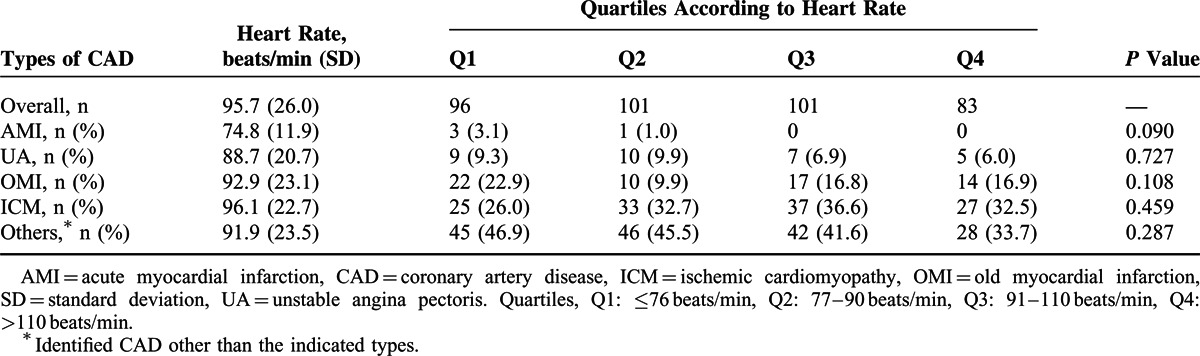
Distributions of Different Types of Coronary Artery Disease

The patients’ clinical outcomes are included in Table 3. During the follow-up period (mean 11 months, maximum 32 months), a higher total mortality rate, including death from both vascular and nonvascular causes, was noted in the CAD group compared with the non-CAD group (21.5% vs 15.5%, *P* = 0.023), but no differences in the incidences of systemic embolism and major bleeding events were noted between the groups. No patients left the study during the follow-up period.

**TABLE 3:**
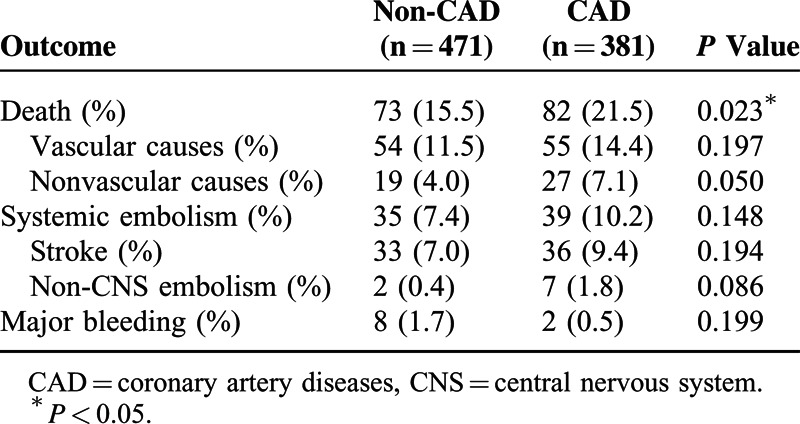
Clinical Outcomes for 1-Year of Follow-up

**FIGURE 1:**
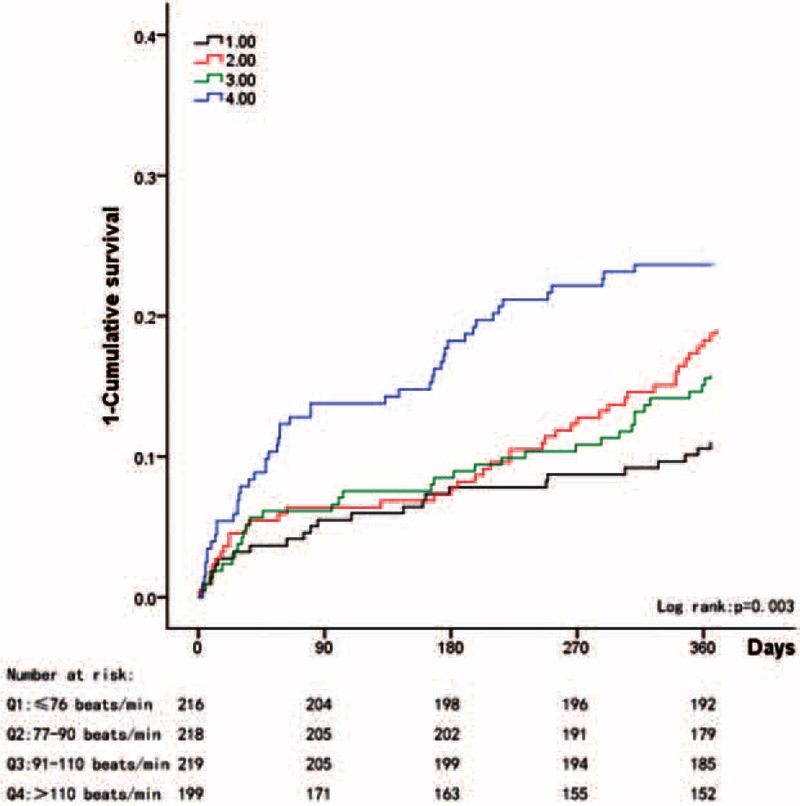
Kaplan–Meier estimates of the 1–cumulative survival rate for unselected patients with permAF.

**FIGURE 2:**
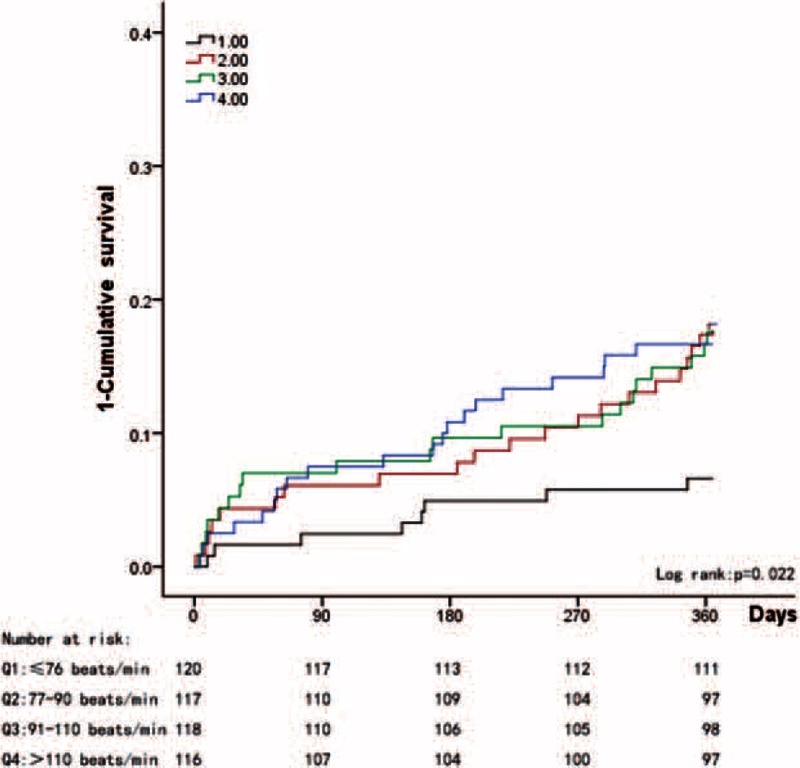
Kaplan–Meier estimates of the 1–cumulative survival rate for patients with permAF without CAD.

**FIGURE 3:**
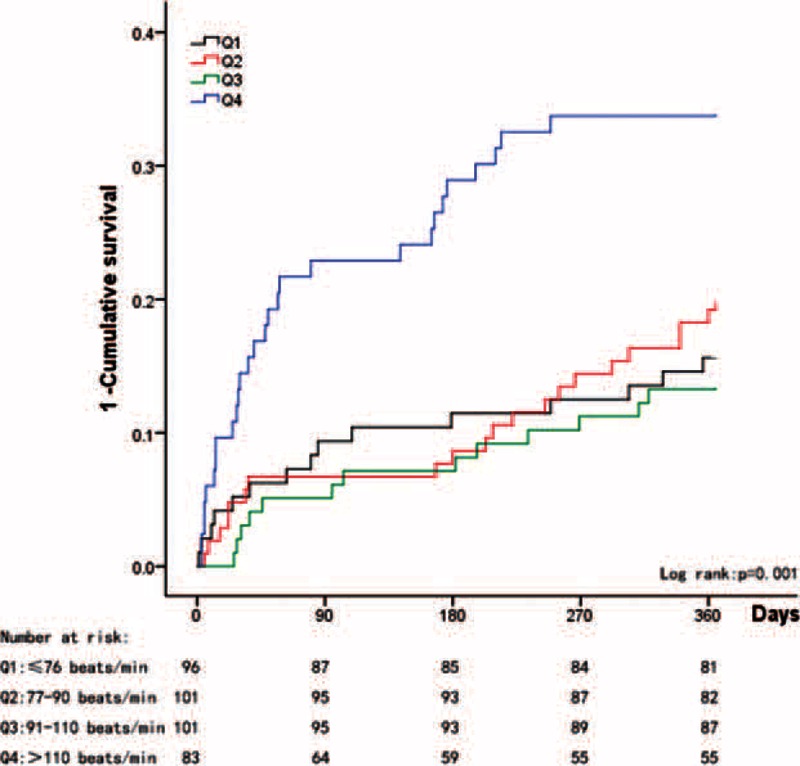
Kaplan–Meier estimates of the 1–cumulative survival rate for patients with permAF and CAD.

When the patients were divided into quartiles according to heart rate (≤76 beats/min, 77–90 beats/min, 91–110 beats/min, and >110 beats/min), significantly different cutoff points were observed between the patients with and without CAD. Among the unselected patients, we noted a gradually increasing mortality rate in conjunction with increases in heart rate (log rank *P* = 0.003); however, similar survival rates were observed between the second and third quartiles (Figure 1). Among the patients without CAD, the lowest quartile (≤76 beats/min) exhibited the best 1-year survival rate (Figure 2); however, among the patients with CAD, the lower 3 quartiles exhibited similar 1-year survival rates, and only the highest quartile (>110 beats/min) exhibited a poor survival rate (Figure 3).

The results of multivariate adjusted Cox regression analysis are presented in Table 4. Age, heart rate, and NYHA classifications were each independently associated with total mortality in both the unselected patients and the patients without CAD. Age, heart rate, and diabetes were each independently associated with total mortality in the patients with CAD. The adjusted HRs for total mortality in the second, third, and fourth quartiles of heart rate compared with those in the first quartile (reference) were 1.738 (95% CI 1.502–2.872; *P* = 0.028),1.621 (95% CI 0.974–2.699; *P* = 0.063), and 2.385 (95% CI 1.461–3.893; *P* = 0.001) in the unselected population; 3.018 (95% CI 1.343–6.786; *P* = 0.008), 3.044 (95% CI 1.359–6.821; *P* = 0.007), and 2.326 (95% CI 1.015–5.332; *P* = 0.046) in the patients without CAD; and 1.127 (95% CI 0.584–2.175, *P* = 0.772), 0.961 (95% CI 0.480–1.922; *P* = 0.910), and 3.001 (95% CI 1.588–5.669; *P* = 0.001) in the patients with CAD. However, heart rate was not associated with systemic embolism among the patients with permAF. Traditional risk factors such as a history of stroke, high blood pressure, and inadequate warfarin use were each independently associated with systemic embolism in the unselected patients. A history of stroke and the use of diuretics were both associated with a lower incidence of systemic embolism among the patients without CAD, whereas female gender, a history of stroke and CCB use were each associated with a higher incidence of systemic embolism among the patients with CAD.

**TABLE 4:**
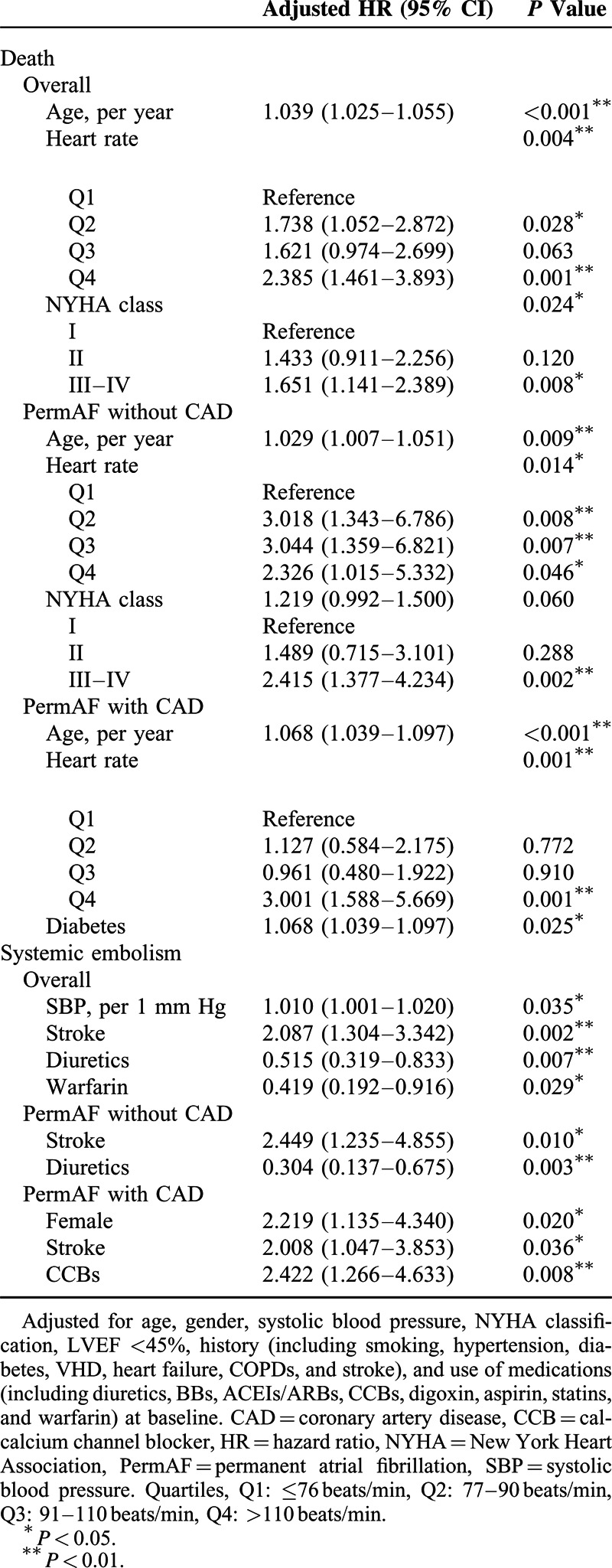
Risk Factors for Death and Systemic Embolism

## DISCUSSION

Our results indicated that there were different baseline characteristics among the patients with permAF, depending on whether the patients in question had CAD. The patients with CAD had more risk factors and comorbidities and a higher mortality rate compared with the patients without CAD. Moreover, the ventricular heart rate was independently associated with mortality among both the unselected patients and the patients without CAD but not among the patients with CAD, unless the heart rate was >110 beats/min.

The relationship between ventricular heart rate and mortality among the patients with permAF in the present study was inconsistent with that noted in previous studies.^3,13,14^ Analysis of the pooled data from both the Rate Control Efficacy in Permanent Atrial Fibrillation (RACE) (28% with CADs) and Atrial Fibrillation Follow-up Investigation of Rhythm Management (AFFIRM) (37% with CADs) studies^14^ indicated that a lower heart rate (<80 beats/min) was not associated with improved clinical endpoints compared with the patients with a heart rate <100 beats/min. The RACE II study^3^ (n = 614, 18.1% with CADs) has been the only prospective randomized controlled trial to investigate whether either “strict” (<80 beats/min) or “lenient” (<110 beats/min) ventricular heart rate control was preferred among patients with permAF. Neither strategy was associated with any significant differences in mortality during the 3-year follow-up period of the study. In another cohort study of permAF and HF (n = 488, 30% with ischemic heart disease), Cullington et al^13^ suggested that slower resting ventricular rates (59–73 beats/min) were not associated with an improved survival rate and that a ventricular rate >90 beats/min did not increase the risk of death. The proportion of patients with a heart rate >110 beats/min was approximately 14% (69/488), which was significantly lower than the percentage noted in our study (25%). Additionally, the usage rates of BBs and digoxin were slightly higher compared with those in our study (52% vs 46%, 51% vs 47%, respectively). Due to the high prevalence of CAD (or ischemic heart disease) and the different ventricular rate distributions observed in these studies, extrapolating their results to the management of patients without CAD would appear to be inappropriate.

A major concern regarding high heart rates is the induction or worsening of ischemia among patients with CAD.^10^ However, this concern was not validated by our observations. A resting heart rate below 110 beats/min was not predictive of mortality in patients with permAF and CAD. Patients with permAF and CAD have more traditional cardiovascular risk factors (aging, male gender, hypertension, and diabetes mellitus) and comorbidities (HF, COPD, and stroke), all of which are predictors of adverse outcomes.^20–22^ These complicated clinical features may obscure the predictive effects of heart rate on mortality. Additionally, AF causes the loss of “atrial kick” as well as reduced LV diastolic filling and decreased stroke volumes. An increase in the ventricular heart rate may compensate for these changes and maintain adequate cardiac output^23^; therefore, lower heart rates, particularly in high-risk patients with CAD and AF, may be hemodynamically detrimental.

Significant differences were observed in the use of medications among the patients with permAF and CAD compared with the patients without CAD. The patients with CAD were more likely to take BBs, CCBs, ACEIs/ARBs, aspirin, and statins. However, digoxin and warfarin were infrequently prescribed in these patients. BBs, CCBs, and digoxin are common rate control medications that work via different mechanisms. BBs, for example, mainly decrease the heart rate when the sympathetic nervous system is activated.^24^ Multiple comorbidities may result in the universal activation of sympathetic tone; therefore, BBs may reduce the differences in heart rate noted in the patients with CAD. According to the Cardiac Insufficiency Bisoprolol Study (CIBIS) II trial (26% with ischemic heart disease)^25^ and the Metoprolol CR/XL Randomized Intervention Trial in Chronic Heart Failure (MERIT-HF) study (32% with previous myocardial infarctions),^26^ BBs do not improve the prognoses of patients with HF and AF and may result in both fatigue and reduced exercise capacity.^24^ Moreover, tight daytime ventricular heart rate control maybe associated with the exacerbation of nocturnal pauses among high-risk patients, which may increase the likelihood of pause-dependent malignant arrhythmia.^27,28^ Additionally, increased aspirin use among patients with CAD and permAF does not reduce the incidence of systemic embolism or improve the survival rate compared with warfarin use.^29,30^

### Limitations

This prospective observational study had several limitations. First, only the patients’ resting ventricular rates were recorded during clinic visits; these rates were different from the patients’ chronic resting heart rates and average heart rates and were usually affected by comorbidities. Second, as this was an observational study, we did not modify the patients’ treatments or repeatedly evaluate their ventricular heart rates during the follow-up period. Third, patients with low ventricular heart rates and regular RR intervals were excluded due to concern regarding atrioventricular block. These patients may have experienced worse outcomes than the patients enrolled in our study. Additionally, stable and unstable CAD may have different effects on the ventricular heart rate among patients with AF; however, we did not distinguish between stable and unstable CAD in the present study. Therefore, the prognostic effects of ventricular heart rate in AF and CAD warrant further study.

## CONCLUSIONS

We observed different cutoff points in the prognostic evaluations among the different populations included in our study. In the patients with permAF without CAD, lower heart rates were indicative of better survival rates, with heart rates ≤76 beats/minute exhibiting the best survival rates. However, this was not the case among the patients with CAD; in these patients, no increase in mortality was observed unless the heart rate was >110 beats/min. This heterogeneity may be partially attributable to the different risk factors and comorbidities that were observed and to the different management strategies that were utilized between the groups. Therefore, among patients with permAF and CAD, more attention should be paid to the management of risk factors and comorbidities than to heart rate control if the heart rate is below 110 beats/min.
